# mFOLFOX6 plus bevacizumab to treat liver-only metastases of colorectal cancer that are unsuitable for upfront resection (TRICC0808): a multicenter phase II trial comprising the final analysis for survival

**DOI:** 10.1007/s10147-018-01393-8

**Published:** 2019-01-05

**Authors:** Masamichi Yasuno, Hiroyuki Uetake, Megumi Ishiguro, Nobuyuki Mizunuma, Takamichi Komori, Go Miyata, Akio Shiomi, Tatsuo Kagimura, Kenichi Sugihara

**Affiliations:** 1 Institute of Global Affairs, Tokyo Medical and Dental University, 1-5-45 Yushima, Bunkyo-ku, Tokyo, 113-8519 Japan; 2 Department of Specialized Surgeries, Tokyo Medical and Dental University, Graduate School, 1-5-45 Yushima, Bunkyo-ku, Tokyo, 113-8519 Japan; 3 Department of Medical Oncology, Cancer Institute Hospital of Japanese Foundation for Cancer Research, 3-8-31 Ariake, Koto, Tokyo, 135-8550 Japan; 4 Department of Surgery, Osaka General Medical Center, 3-1-56 Bandai-Higashi, Sumiyoshi, Osaka, 558-8558 Japan; 5 Department of Surgery, Iwate Prefectural Central Hospital, 1-4-1 Ueda, Morioka, Iwate, 020-0066 Japan; 6 Colon and Rectal Surgery, Shizuoka Cancer Center, 1007 Shimonogakubo, Nagaizumi, Shunto, Shuzuoka, 411-8777 Japan; 7 Department of Statistical Analysis, Translational Research Informatics Center, 1-5-4 Minatojima-minamimachi, Chou, Kobe, Hyogo 650-0047 Japan; 8 Tokyo Medical and Dental University, Graduate School, 1-5-45 Yushima, Bunkyo-ku, Tokyo, 113-8519 Japan

**Keywords:** Liver metastases, Colorectal cancer, Initially resectable, Initially unresectable, Hepatectomy

## Abstract

**Background:**

The TRICC0808 trial is a phase II multi-institutional trial that investigated the efficacy of preoperative mFOLFOX6 + bevacizumab (BV) therapy for liver-only metastasis that is unsuitable for upfront resection. The R0 resection rate in the efficacy analysis has been reported to be 44.4%, and the final analysis for survival was conducted (data fixation on February 16, 2015).

**Methods:**

Six cycles of mFOLFOX6 + BV therapy were applied to patients with liver-only metastases, which were > 5 cm in diameter or more than four tumors (H2 and H3), and hepatectomy was performed if possible. Primary and secondary endpoints were the R0 hepatectomy rate and overall survival (OS), respectively.

**Results:**

Of 46 patients registered, OS was analyzed for 45 patients in whom the 3-year OS rate from the starting date of chemotherapy was 44.0% with a 33.6-month median survival time (MST). The 3-year OS rate of 31 patients with hepatectomy, including resection after an additional chemotherapy, was 61.3% with a 43.1-month MST, which was significantly better than 0% of the 3-year OS rate with a 21.0-month MST of 14 patients without hepatectomy (*p* value < 0.0001). In 24 patients who underwent hepatectomy after six cycles of protocol chemotherapy, the 3-year relapse-free survival rate was 8.3%, with a 36.8-month MST.

**Conclusions:**

This final analysis of the TRICC0808 trial revealed a better long-term survival in patients with hepatectomy after mFOLFOX6 + BV therapy, although most examined patients eventually developed recurrence. Thus, hepatectomy after chemotherapy might improve the survival in patients with advanced liver metastases, although cure remains difficult.

**Electronic supplementary material:**

The online version of this article (10.1007/s10147-018-01393-8) contains supplementary material, which is available to authorized users.

## Introduction

The liver is a frequent site of metastases from colorectal cancer [1–3], and hepatectomy is the only curative treatment, with a 5-year survival rate of 40–60% [3–7]. However, only a minority of patients with liver metastases are initially eligible for radical hepatectomy [8]. Reportedly, the initial resection rate is reported to be < 25% because of the higher number and size and the complicated location of tumor [9, 10]. In patients with multiple or large liver metastases, liver tumors might not be completely resected or recurrence even after R0 hepatectomy is frequently observed. In addition, chemotherapies with new potent cytotoxic drugs, such as irinotecan and oxaliplatin [11], and molecule-targeted drugs [12–16] have improved the response rate for metastatic colorectal cancer. In the treatment of patients with unresectable liver metastases, conversion therapy has been applied to decrease the tumor size and facilitate resection via preoperative chemotherapy with new potent drugs [17–19].

Thus, we conducted a phase II multi-institutional clinical study—TRICC0808—to investigate the efficacy and safety of preoperative modified oxaliplatin-infused 5-fluorouracil leucovorin (mFOLFOX6) + bevacizumab (BV) therapy in patients with colorectal cancer and liver-only metastases in whom upfront surgery was unsuitable (maximum metastatic focal diameter, > 5 cm or number of metastases, ≥ 5; H2/H3) [20, 21]. Previously, we reported that mFOLFOX6 + BV yielded R0 hepatectomy rate of 44.4% and conversion rate of 23.1%, whereas the rate of surgical complications was low. This study presented the final survival data analysis of TRICC0808 after the 3-year follow-up from the start date of mFOLFOX6 + BV therapy (final data fixation was on February 16, 2015).

## Patients and methods

### Study design

TRICC0808 was a multicenter, phase II clinical study; details of the trial design and study procedures are available elsewhere [21]. The primary trial endpoint was the R0 hepatectomy rate for the entire study population, and secondary endpoints were the R0 hepatectomy rate relative to all resected cases, the percentage of initially resectable and subsequently unresectable cases, the percentage of initially unresectable and subsequently resectable cases, hepatectomy safety, recurrence rate, and survival period. The primary endpoint and treatment results of chemotherapy are provided elsewhere [21].

### Patients

Patients eligible for this study were as follows: those aged 20–80 years; those with an Eastern Cooperative Oncology Group Performance Status (ECOG PS) of 0–1; those with histologically proven colorectal cancer; those with maximum liver metastatic focal diameter of > 5 cm or with more than five liver metastases (H2/H3); those who underwent curative primary tumor resection ≥ 28 days before registration; those with no detectable extrahepatic metastases; those with no history of chemotherapy. This study protocol was approved by the medical ethics committees of all participating facilities, and written informed consent was obtained from all patients before registration.

### Procedures

Within 2 weeks of registration, each registered patient received the first mFOLFOX6 + BV therapy. After three treatment cycles, the first diagnostic imaging evaluation was performed. For patients in whom the diagnostic imaging evaluation was progressive disease (PD) or stable disease (SD), tumor growth might make resection impossible; therefore, in such patients, hepatectomy was performed if feasible or the protocol treatment was discontinued. In patients rated as complete response (CR), partial response (PR), or SD, mFOLFOX6 + BV therapy was administered for three additional cycles (during the sixth cycle, only mFOLFOX6 was administered). After six cycles, the second diagnostic imaging evaluation was performed. If deemed resectable, then hepatectomy was performed, whereas if deemed unresectable, then the protocol treatment was immediately discontinued.

R0 hepatectomy was defined by the absence of microscopic tumor invasion of the resection margins, R1 hepatectomy was defined by the presence of microscopic tumor invasion at the resection margins, and R2 hepatectomy was defined by the presence of macroscopic residual tumor [20]. Patients who underwent thermal coagulation therapy, radiofrequency ablation (RFA), or microwave coagulation therapy were considered for R2 hepatectomy. In addition, patients who underwent R1 or R2 hepatectomy were treated further at physicians’ discretion. Postoperative adjuvant chemotherapy was not routinely administered after R0 hepatectomy.

The pathological response of chemotherapy was evaluated in resected specimens based on the JSCCR criteria [20]. Patients were followed up every 6 months with contrast-enhanced computed tomography (CT) and the measurement of serum CEA level. Overall survival (OS) was defined as the time interval between the starting date of chemotherapy and the date of death. Furthermore, patients who underwent hepatectomy were assessed for relapse-free survival (RFS) and OS at 1-, 2-, and 3-year postoperatively by clinical examinations, including tumor markers, and contrast-enhanced CT.

### Statistical analysis

The sample size calculation and null and alternative hypotheses about the primary endpoint have been reported previously [21]. OS and RFS rates were estimated using the Kaplan–Meier method and compared by the log-rank test. Groups were compared with the use of Fisher’s exact test or the Chi-square test for categorical variables and the Wilcoxon signed-rank test for continuous variables. In this study, statistical analyses were conducted using SAS version 9.3 (SAS Institute Inc., Cary, NC), and statistical significance was set at the 0.05 level.

## Results

From April 2009 to October 2011, 46 patients from 25 institutes were enrolled. However, one patient was found ineligible after commencing the treatment. The median follow-up period after the start of protocol chemotherapy was 31.4 (range 10.1–63.7) months.

### Patients’ characteristics

All eligible patients underwent curative primary tumor resection ≥ 28 days before registration. The median number of liver metastases was 6 (range 1–23). The reason for being unresectable was the inadequate volume of the remnant liver in most patients (88.5%) and poor anatomical localization of tumor in a few patients (7.7%) (Supplemental Table 1).

### Protocol chemotherapy and hepatectomy

Forty-one patients completed the planned six cycles of protocol chemotherapy (chemotherapy completion rate, 91.1%) (Supplemental Fig. 1). Hepatectomy was performed after six cycles of the protocol treatment in 24 (53.3%) patients, with R0 hepatectomy in 20 and R1/R2 hepatectomy in four (including three patients judged as R2 for the additional RFA). Most surgical procedures were major hepatectomy (54.1%) that were hepatic resection of three or more adjacent Couinaud segments. Of 19 patients with initially resectable liver metastases, 18 (94.7%) underwent hepatectomy (R0, 16; R1/R2, 2). Of 26 initially unresectable patients, six (23.1%) underwent hepatectomy after the protocol treatment (R0, 4; R1/R2, 2), and therefore, the conversion rate was 23.1%. No surgery-related deaths or any second surgery owing to complications was required in this study population.

Chemotherapies after hepatectomy were performed in 10 (42%) of 24 patients (FOLFOX, 2; mFOLFOX6 + BV, 2; mFOLFOX6, 5; CapeOX, 1; uracil + tegafur/leucovorin, 1).

### Chemotherapy after post-protocol treatment and hepatectomy

Post-protocol chemotherapies were performed in 18 unresectable patients [mFOLFOX6/capecitabine + oxaliplatin (CapeOX) ± BV, 15; folinic acid, fluorourcil, and irinotecan (FOLFIRI) ± BV/cetuximab, 8; irinotecan + S-1 (IRIS) ± BV, 2; simplified biweekly leucovorin and fluorouracil (sLV5FU2) + BV, 1; hepatic arterial infusion (HAI), 1]. Of these, seven patients underwent hepatectomy. Regimens of post-protocol chemotherapy in patients undergoing hepatectomy were four cycles of mFOLFOX6 + BV plus seven cycles of FOLFIRI + panitumumab, three cycles of mFOLFOX6 + BV, six cycles of mFOLFOX6 + BV, one cycle of mFOLFOX6 + BV plus four cycles of FOLFIRI + BV, one cycle of FOLFIRI, six cycles of mFOLFOX6 + BV plus 3 years of sLV5FU2 + BV, six cycles of mFOLFOX6 + BV, and 22 cycles of IRIS/IRIS + S-1 plus FOLFIRI + BV.

### Survival

**Fig. 1:**
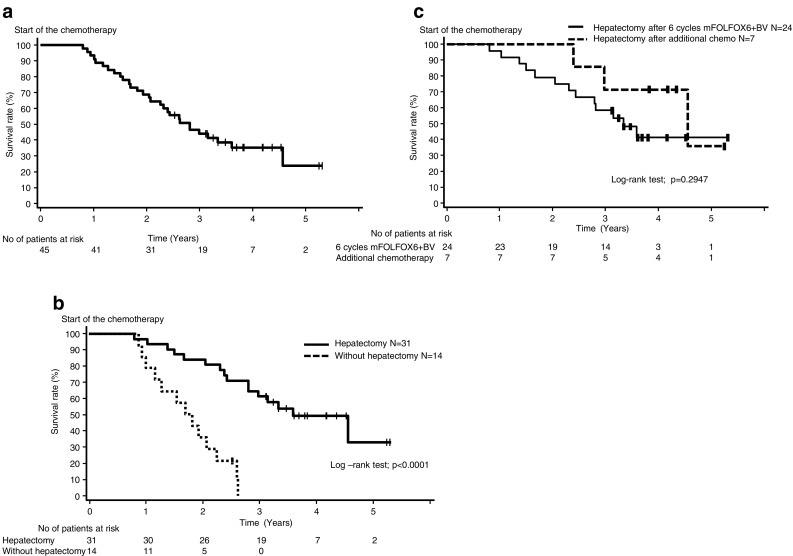
Overall survival from start of mFOLFOX6 + BV therapy. **a** All eligible patients (*N* = 45). **b** Patients with hepatectomy including surgery after post-protocol chemotherapy (solid line, *N* = 31), Patients without hepatectomy (dashed line, *N* = 14). **c** Patients with hepatectomy after protocol 6 cycles mFOLFOX 6 + BV (solid line, *N* = 24); patients with hepatectomy after post-protocol additional chemotherapy (dashed line, *N* = 7)

The 1-, 2-, and 3-year OS rates in 45 eligible patients were 91.1% (95% CI 78.0–96.0%), 68.9% (95% CI 53.2–80.2%), and 44.0% (95% CI 29.2–57.8%), respectively, with 33.6 (95% CI 24.7–43.1) months of the MST (Fig. 1a). The 3-year OS rate in 31 patients with hepatectomy, including seven who underwent surgery after additional chemotherapy, was 61.3% (95% CI 42.0–75.8%), with the 43.1-month MST (95% CI 33.6–not reached), which was better than 0% of the 3-year OS rate with the 21.0-month MST (95% CI 11.9–27.0 months) in patients without hepatectomy (*p* value < 0.0001; Fig. 1b). No differences were observed in the survival time between 24 patients with hepatectomy after six cycles of protocol chemotherapy and seven patients with hepatectomy after an additional chemotherapy (*p* value = 0.2947; Fig. 1c).

**Fig. 2:**
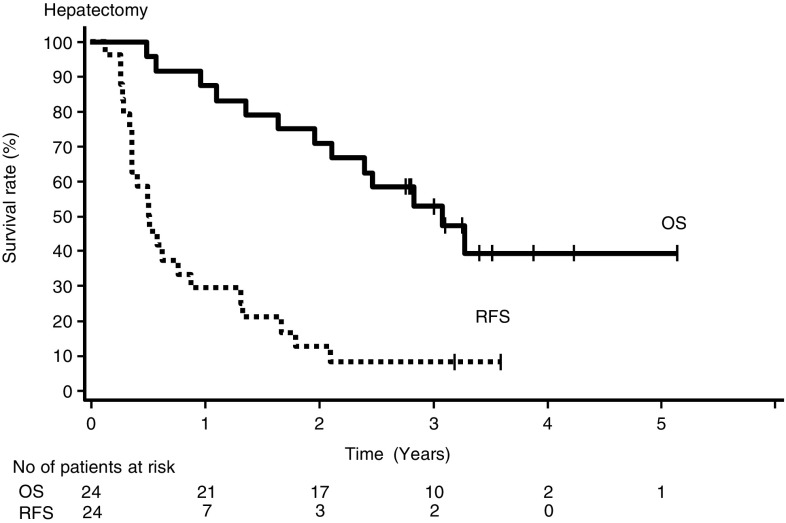
Survival from hepatectomy in patients with surgery after 6 cycles protocol mFOLFOX 6 + BV therapy; overall survival (OS, solid line); relapse-free survival (RFS, dashed line)

In 24 patients with hepatectomy after six cycles of protocol chemotherapy, the 1-, 2-, and 3-year OS rates were 87.5% (95% CI 66.1–95.5%), 70.8% (95% CI 48.4–84.9%), and 53.0% (95% CI 31.2–70.8%), respectively, with the 36.8-month MST (95% CI 23.5–not reached), and the 1-, 2-, and 3-year RFS rates were 29.2% (95% CI 13.0–47.6%), 12.5% (95% CI 3.1–28.7%), and 8.3% (95% CI 1.4–23.3%), respectively, with the 5.9 months of median RFS (95% CI 4.1–10.2 months; Fig. 2). MST and median RFS in 20 patients with R0 hepatectomy were 39.2 (95% CI 23.5–not reached) and 6.2 months (95% CI 4.1–15.2 months), respectively. Among patients with R0 hepatectomy, the 3-year OS rates of 16 patients with resectable liver metastases at registration and four with unresectable liver metastases at registration were similar.

Tables 1 and 2 present clinicopathological factors and survival of eligible patients and 24 patients with hepatectomy after six cycles of protocol chemotherapy. In all eligible patients, the objective response to chemotherapy and presence of hepatectomy exhibited a markedly better OS, whereas T4a/T4b primary tumors exhibited a worse prognosis. In the 24 patients with hepatectomy, the survival period was not considerably different among the degrees of pathological response.

**Table 1:** Overall survival analysis in all eligible cases

	*N*	Overall survival		MST (months)	Log-rank test
		1 year (%)	3 years (%)		*p* value
Sex					
Male	26	92.3	46.2	33.6	0.7737
Female	19	89.5	40.9	33.6	
Location of primary tumor					
Colon	31	90.3	31.1	29.2	0.1038
Rectosigmoid	7	100.0	57.1	43.1	
Rectum	7	85.7	85.7	–	
Lymph node of primary tumor					
N0	12	91.7	48.6	31.4	0.2982
N+	33	90.9	42.4	33.6	
Depth of primary tumor					
T1/T2	2	100.0	50.0	–	0.0250
T3	19	94.7	52.6	54.7	
T4a	18	88.9	48.9	31.4	
T4b	6	83.8	0.0	20.8	
Timing of liver metastases					
Synchronous	39	92.3	43.6	33.6	0.9552
Metachronous	6	83.3	50.0		
Degree of liver metastases					
H2	31	90.3	47.5	35.6	0.1880
H3	14	92.9	35.7	23.3	
Number of liver metastases					
1–4	12	91.7	50.0	–	0.4818
5–10	23	87.0	38.3	29.2	
11 ≤	10	100.0	50.0	37.2	
Maximal size of liver metastases					
≤ 5 cm	19	89.5	45.6	35.6	0.7220
5 cm <	26	92.3	42.3	28.9	
Overall response					
PR	25	100.0	48.0	33.6	< 0.0001
SD	17	88.2	41.2	31.2	
PD	2	0.0	0.0	10.8	
N.E	1	100.0	–	–	
Initial resectability					
Resectable	19	94.7	67.7	53.3	0.0103
Unresectable	26	88.5	26.9	11.5	
Hepatectomy					
Resection	31	96.8	61.3	43.1	< 0.0001
Without resection	14	78.6	0.0	21.0	

*MST* median survival time, *PR* partial response, *SD* stable disease, *PD* progressive disease, *NE* not evaluated, *CI* confidence interval

**Table 2:** Overall survival in the patients with hepatectomy after protocol treatment

	*N*	Overall survival		MST (months)	Log-rank test
		1 year (%)	3 years (%)		*p* value
Timing of liver metastases					
Synchronous	20	85.0	53.3	36.8	0.9467
Metachronous	4	100.0	50.0	–	
Degree of liver metastases					
H2	17	94.1	56.6	36.8	0.2933
H3	7	71.4	42.9	25.3	
Number of liver metastases					
1–4	9	88.9	55.6	–	0.9250
5–10	14	85.7	49.0	34.0	
11 ≤	1	100.0	100.0	39.2	
Maximal size of liver metastases					
≤ 5 cm	8	100.0	60.0	36.8	0.5781
5 cm <	16	81.3	50.0	34.0	
Overall response					
PR	18	88.9	48.6	34.0	0.4600
SD	6	83.3	66.7	–	
Initial resectability					
Resectable	18	94.4	59.3	–	0.0864
Unresectable	6	66.7	33.3	19.8	
Hepatectomy					
Wedge	11	90.9	45.5	29.5	0.5566
Major	13	84.6	59.3	39.2	
Pathological response^a^					
Grade 1a	9	77.8	44.4	29.5	0.8704
Grade 1b	6	83.3	50.0	–	
Grade 2	7	100.0	53.6	39.2	
Grade 3	1	100.0	100.0	–	
Unknown	1	100.0	100.0	36.8	
Completeness of hepatectomy					
R0	20	90.0	53.3	39.2	0.4283
R1/R2	4	75.0	50.0	26.5	
Adjuvant chemotherapy after hepatectomy					
Yes	10	100.0	50.0	36.6	0.9526
No	14	78.6	57.1	36.8	

^a^Pathological response: grade 0, with no necrosis or cellular or structural change; grade 1a; with necrosis or disappearance of tumor in < 1/3 of the entire tumor, grade 1b; with necrosis or disappearance of the tumor in < 2/3 of the entire tumor, grade 2; with necrosis or disappearance of the tumor in > 2/3 of the entire tumor but with viable tumor cells remaining, and grade 3; with the entire tumor presenting necrosis and/or fibrosis and no viable tumor cells identified

### Recurrence

Recurrence was observed in 16 (80%) of 20 patients with R0 hepatectomy. All of four patients with R1/R2 hepatectomy developed remnant liver limited recurrence early after surgery. The median RFS after hepatectomy was 5.3 months. In addition, 14 (58.3%) patients developed recurrence in the liver, including 11 (45.8%) with liver-only diseases and 3 (12.5%) with liver diseases plus lung metastases (Table 3). Furthermore, surgeries for recurrent diseases were undergone in eight (40%) of 20 patients with recurrence, including five (45.5%) of 11 patients with liver-only recurrence and three (100%) of three patients with lung-only recurrence (Table 3).

**Table 3:** Recurrence after hepatectomy

	Hepatectomy after 6 cycles protocol chemotherapy	Hepatectomy after additional chemotherapy
	*N* = 24 (R0; 20, R1/R2; 4)	*N* = 7
	*N* (%)	
Recurrence after hepatectomy		
Yes	20 (83.3%)	6 (85.7%)
No	4 (16.7%)	1 (14.3%)
First site of recurrent disease		
Liver	14 (58.3%)	4 (83.3%)
Lung	7 (29.2%)	0
Lymph node	3 (12.5%)	2 (28.6%)
Others (brain, adrenal)	4 (16.7%)	1 (14.3%)
Disease-free interval after hepatectomy		
Median (months)	5.32	4.62
Treatment after recurrence		
Surgery (radical resection)	8 (33.3%)	1 (14.3%)
Chemotherapy	9 (37.5%)	5 (71.4%)
Others (radiotherapy)	3 (12.5%)	1 (14.3%)
Palliative therapy	2 (8.3%)	0

## Discussion

Several studies have investigated combination therapy with surgery and preoperative chemotherapy for resectable metastases to improve the R0 rate and for unresectable metastases to convert to a resectable status (conversion). However, several of these studies were retrospective or subgroup analysis in randomized trials, and randomized, controlled studies designed to assess conversion chemotherapy were limited [17–19, 22–26]. Thus, the optimal treatment approach for patients who are unsuitable for upfront resection liver metastases remains unclear.

Previously, the TRICC0808 study illustrated that preoperative mFOLFOX6 + BV therapy attained a high R0 hepatectomy rate (44.4%) and conversion rate (23.1%) for patients with liver metastases who were unsuitable for upfront resection [21]. As the R0 rate was approximately 45% of the anticipated rate, it fulfilled the primary endpoint. In addition, compared with the previous studies for patients who were unsuitable for upfront resection, the R0 and conversion rates in this study were encouraging [17–19, 23, 24]. This final survival analysis of TRIC0808 demonstrated that hepatectomy following mFOLFOX6 + BV therapy attained favorably extended survival. Precisely, 24 patients with hepatectomy after protocol chemotherapy attained 36.8 months of MST, and MST in all 31 patients who underwent hepatectomy, including seven patients after an additional chemotherapy, was 43.1 months, although most patients had surgical difficulties and poor prognostic factors [27]. However, disease recurrence occurred early after the surgery, with 5.3 months of median RFS, in patients with hepatectomy after protocol therapy.

We evaluated the associations of clinicopathological factors with OS (Tables 2, 1). The size and number were not prognostic factors, since most patients had numerous (median number, 6) and large (median size, 5.3 cm) hepatic metastases.

Patients with T4a/T4b primary tumor exhibited worse prognosis, although hepatectomy rate was similar to that of patients with T1/T2/T3 tumor. Patients with T4a/T4b developed only remnant liver limited recurrence.

In this trial, 68.9% of patients had colon cancer. Patients with colon cancer had a lower hepatectomy rate and worse OS compared with rectosigmoid/rectum cases (no statistical difference). Recently, primary tumor location (sidedness) has been reported to be a critical prognostic factor in metastatic colorectal cancer [28]. Regrettably, we did not collect information on sidedness of colon cancer; therefore, we were unable to do subset analysis for sidedness.

Previously, the pathological response to preoperative chemotherapy in liver metastases has been reported to correlate with a favorable survival outcome [18, 29]. However, in this study, the pathological response exhibited no marked impact on the survival, which may have been because of the small number of examined patients.

The CELIM study was a well-planned, prospective study investigating the efficacy of preoperative chemotherapy (FOLFOX or FOLFIRI plus cetuximab) for patients who were unsuitable for upfront resection liver metastases, which were defined as technically unresectable and/or more than five liver tumors [19, 30]. Although the entry criteria and definition of resectability were similar to our study, it slightly differed, which necessitated cautious and direct comparison. A similar number of patients recruited for hepatectomy (R0 rate; TRICC0808 44% vs. CELIM 34%) and a similar number of initially technically unresectable patients proceeded to hepatectomy (conversion rate; TRICC0808 23% vs. CELIM 28%). Regarding the survival, MST in all eligible patients was similar in both studies (TRICC0808 study, 33.6 months; CELIM study, 35.7 months). In contrast, for patients who underwent R0 hepatectomy, the MST of 39.2 months in this study is worse than 53.9 months reported in the CELIM study [30]. In both studies, disease recurrences occurred early after surgery in most patients; however, 6.2 months of the median RFS in this study was worse than 9.9 months in the CELIM study. A significant portion of patients enrolled in this study had unfavorable prognostic factors [27]; that is, 34% of our patients who underwent hepatectomy had massive disease infiltration (H3; ≤ 5 lesions and > 5 cm liver metastases) and 83% of patients had a synchronous metastatic tumor. Despite the limits of comparing the results of different studies, our survival results seem encouraging.

The previous studies have reported that patients with initially unresectable liver metastases, whose metastases were rendered resectable after chemotherapy and underwent hepatectomy, exhibited a high recurrence rate (> 70%) and short median RFS (16–19 months) [4, 31] but better OS [32, 33]. In addition, patients with postoperative recurrence of metastatic disease would likely benefit from an additional surgery or second-line chemotherapy [34], which might explain why patients exhibited a relatively prolonged survival, considering the early recurrences after hepatectomy in our study and in the other studies. However, other studies did not provide details of the recurrence and treatment for recurrent diseases. In this study, among 20 patients who developed recurrence after hepatectomy, eight (40%) underwent radical resection for recurrent tumors and nine (45%) proceeded to the second-line chemotherapy for recurrent diseases (Table 3), which might explain why patients in this study exhibited a relatively prolonged survival, considering the early recurrences after hepatectomy.

In patients who are unsuitable for upfront resection liver metastases, a regimen resulting in high response and large-size reduction is recommended [35]. According to a consensus statement for downsizing, FOLFOX and FOLFIRI are two standard chemotherapy techniques with equivalent efficacy [36]. Regarding biological agents, a recent meta-analysis concluded that the addition of an anti-EGFR antibody to chemotherapy markedly increases the response and R0 hepatectomy rate, whereas the addition of BV exhibits no benefit in tumor regression [13]. Furthermore, cetuximab plus FOLFOX or FOLFIRI has shown to improve both resectability and survival in patients with Kras wild-type liver metastases [25].

The ESMO guideline [37] states that only a few trials specifically designed to investigate “conversion” chemotherapy were randomized, controlled trials, making it difficult to reach any decision regarding the “best” regimen to use in this clinical setting. Seemingly, a combination of cytotoxic doublet and an anti-EGFR antibody has the best benefit risk/ratio in patients with RAS wild-type disease, although the combination of FOLFOXIRI plus BV might also be considered and to a lesser extent, a cytotoxic doublet plus BV.

In patients with an RAS-mutant disease, a cytotoxic doublet plus BV or fluorouracil, leucovorin, oxaliplatin, and irinotecan (FOLFOXIRI) plus BV is recommended. Tumor RAS status should be determined for every case of metastatic colorectal cancer. However, the Japanese social insurance system did not include RAS mutation testing until 2010 after we started this trial. We, therefore, chose mFOLFOX6 + BV as protocol regimen and did not investigate RAS status.

The combined use of BV with FOLFOX or FOLFIRI is not compelling in the preoperative setting. However, the combined use of BV provides a higher response rate and prolonged survival in patients with both Kras wild-type and mutant [38]. Reportedly, chemotherapeutic agents might cause liver injuries, such as steatohepatitis and sinusoidal congestion, and surgical complications [39]. In contrast, the combined use of BV and oxaliplatin can decrease sinusoidal congestion [40]. In this study, a high radiological response rate (55.6%) and pathological response rate (major response with necrosis of > 1/3 of the tumor; 42.5%) were observed. However, the rate of surgical complication was low. Based on these perspectives, the combined use of BV and oxaliplatin is acceptable for patients who are unsuitable for upfront resection liver metastases, which are not confined to the Kras wild-type population.

In patients with extensive liver involvement, mFOLFOX6 + BV preoperative therapy has proven to be a promising first-line treatment to improve the R0 rate and for conversion. MST of 43.1 months in the patients with hepatectomy is much longer than what could be expected with chemotherapy alone; however, MST of 33.6 months in all eligible patients might not be enough for including 69% of the patients with hepatectomy. Synchronous liver metastases with major liver involvement were usually characterized as having a very poor prognosis [27]. In our study, 87% of patients had a synchronous metastatic tumor and 31% of patients had massive disease infiltration (H3; 5 ≤ lesions and 5 cm < size). Therefore, survival in unresectable patients was very poor, and disease recurrences occurred early after hepatectomy in most patients. The survival benefit of preoperative mFOLFOX6 + BV therapy for patients with very poor prognosis factors might still be controversial.

## Conclusion

Preoperative mFOLFOX6 + BV therapy for patients who are unsuitable for upfront resection liver metastases from colorectal cancer could contribute to the better survival outcome by increasing the number of patients undergoing R0 hepatectomy. However, most patients in this study eventually developed recurrence. Regarding the treatment of disease, clinical benefits of preoperative chemotherapy for patients who are unsuitable for upfront resection colorectal liver metastases might still be controversial.

## Electronic supplementary material

Below is the link to the electronic supplementary material.

Supplementary material 1 (DOCX 114 KB)

**Supplemental Figure 1**. Trial progress. **Abbreviations**; CR, complete response; NE, not evaluated; PD, progressive disease; PR, partial response; SD, stable disease. *: Hepatectomy after 4 cycle therapy (PPTX 51 KB)
