# Supplementary material for: mFOLFOX6 plus bevacizumab to treat liver-only metastases of colorectal cancer that are unsuitable for upfront resection (TRICC0808): a multicenter phase II trial comprising the final analysis for survival
Source: Int J Clin Oncol. 2019 Jan 5;24(5):516–25. doi: 10.1007/s10147-018-01393-8 (PMC6469677; doi:10.1007/s10147-018-01393-8)
Supplement: Supplementary file 1 — Supplementary material 1 (DOCX 114 KB) [file 10147_2018_1393_MOESM1_ESM.docx]

**Supplemental Table 1.** Patient characteristics

*; P=0.0137 between resectable and unresectable, +; Major vascular invasion or poor location
