# Supplementary material for: mFOLFOX6 plus bevacizumab to treat liver-only metastases of colorectal cancer that are unsuitable for upfront resection (TRICC0808): a multicenter phase II trial comprising the final analysis for survival
Source: Int J Clin Oncol. 2019 Jan 5;24(5):516–25. doi: 10.1007/s10147-018-01393-8 (PMC6469677; doi:10.1007/s10147-018-01393-8)
Supplement: Supplementary file 2 — Supplemental Figure 1. Trial progress. Abbreviations; CR, complete response; NE, not evaluated; PD, progressive disease; PR, partial response; SD, stable disease. *: Hepatectomy after 4 cycle therapy (PPTX 51 KB) [file 10147_2018_1393_MOESM2_ESM.pptx]

## Slide 1
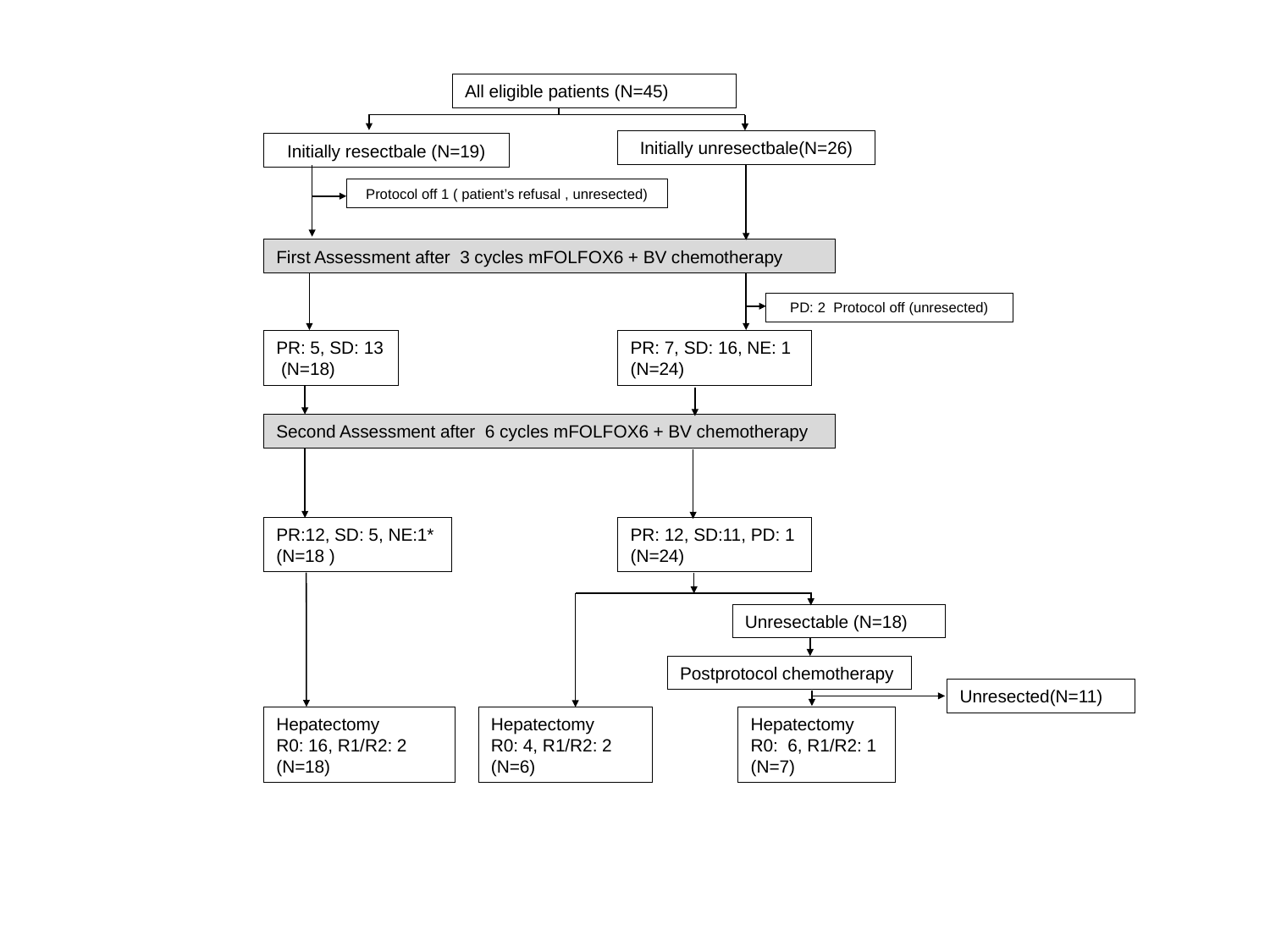

All eligible patients (N=45)
Initially unresectbale(N=26)
Initially resectbale (N=19)
Protocol off 1 ( patient’s refusal , unresected)
First Assessment after 3 cycles mFOLFOX6 + BV chemotherapy
PD: 2 Protocol off (unresected)
PR: 5, SD: 13
 (N=18)
PR: 7, SD: 16, NE: 1
(N=24)
Second Assessment after 6 cycles mFOLFOX6 + BV chemotherapy
PR:12, SD: 5, NE:1*
(N=18 )
PR: 12, SD:11, PD: 1
(N=24)
Unresectable (N=18)
Postprotocol chemotherapy
Unresected(N=11)
Hepatectomy
R0: 16, R1/R2: 2
(N=18)
Hepatectomy
R0: 4, R1/R2: 2
(N=6)
Hepatectomy
R0: 6, R1/R2: 1
(N=7)
